# Manufacturing a chimpanzee adenovirus‐vectored SARS‐CoV‐2 vaccine to meet global needs

**DOI:** 10.1002/bit.27945

**Published:** 2021-11-15

**Authors:** Carina C. D. Joe, Jinlin Jiang, Thomas Linke, Yuanyuan Li, Sofiya Fedosyuk, Gaurav Gupta, Adam Berg, Rameswara R. Segireddy, David Mainwaring, Amar Joshi, Paul Cashen, Byron Rees, Nitin Chopra, Piergiuseppe Nestola, Jonathan Humphreys, Sarah Davies, Nick Smith, Scott Bruce, Dennis Verbart, Daan Bormans, Carol Knevelman, Mark Woodyer, Lee Davies, Lisa Cooper, Maria Kapanidou, Nicole Bleckwenn, Daniel Pappas, Teresa Lambe, Daniel C. Smith, Catherine M. Green, Raghavan Venkat, Adam J. Ritchie, Sarah C. Gilbert, Richard Turner, Alexander D. Douglas

**Affiliations:** ^1^ Nuffield Department of Medicine, Jenner Institute University of Oxford Oxford UK; ^2^ Biopharmaceuticals Development R&D, AstraZeneca Gaithersburg Maryland USA; ^3^ Pall Europe Portsmouth UK; ^4^ Sartorius Stedim Biotech GmbH Goettingen Germany; ^5^ Sartorius Stedim Switzerland AG Tagelswangen Switzerland; ^6^ Vaccine Manufacturing and Innovation Centre Oxford UK; ^7^ Cobra Biologics Keele UK; ^8^ Halix BV Leiden The Netherlands; ^9^ Oxford Biomedica PLC Cowley UK; ^10^ Clinical Biomanufacturing Facility, Nuffield Department of Medicine University of Oxford Oxford UK; ^11^ Purification Process Sciences, Biopharmaceuticals Development R&D, AstraZeneca Cambridge UK

**Keywords:** adenovirus, distributed manufacturing, vaccine

## Abstract

Manufacturing has been the key factor limiting rollout of vaccination during the COVID‐19 pandemic, requiring rapid development and large‐scale implementation of novel manufacturing technologies. ChAdOx1 nCoV‐19 (AZD1222, Vaxzevria) is an efficacious vaccine against SARS‐CoV‐2, based upon an adenovirus vector. We describe the development of a process for the production of this vaccine and others based upon the same platform, including novel features to facilitate very large‐scale production. We discuss the process economics and the “distributed manufacturing” approach we have taken to provide the vaccine at globally‐relevant scale and with international security of supply. Together, these approaches have enabled the largest viral vector manufacturing campaign to date, providing a substantial proportion of global COVID‐19 vaccine supply at low cost.

## Graphical abstract

Well over a billion doses of the ChAdOx1 nCoV‐19 vaccine developed by The University of Oxford and AstraZeneca have been produced and distributed at low cost. This represents a substantial proportion of global COVID‐19 vaccine supply to date, especially in low‐ and middle‐income countries. The authors report on the development of the manufacturing process for the vaccine, and the process economics and distributed manufacturing approach involving bulk vaccine (drug substance) production on five continents.

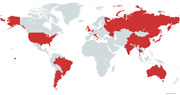

## 1 INTRODUCTION

Adenovirus vectors are among the leading SARS‐CoV‐2 vaccines. The use of simian adenoviruses circumvents pre‐existing immunity to common human serotypes (Ewer et al., 2017). ChAdOx1 nCoV‐19 (also known as AZD1222) has been developed by the University of Oxford in partnership with AstraZeneca, and has demonstrated efficacy against SARS‐CoV‐2 infection (Voysey et al., 2020). The product has been supplied for use in more than 170 countries (“Tracking Coronavirus Vaccinations Around the World,” 2021). AstraZeneca (AZ) and its partner the Serum Institute of India (SII) aim to supply 3 billion doses of 5 × 10^10^ virus particles (VP) in 2021, including as the biggest suppliers to the Gavi‐led COVAX initiative to promote global equity of access to SARS‐CoV‐2 vaccines (Gavi, 2021).

This supply commitment entails the production of nearly 2 × 10^20^ VP of bulk drug substance (DS). To our knowledge the greatest previously‐disclosed scale of manufacture of adenovirus vectors for human use was the production of c. 2 × 10^17^ VP (for 2.7 million doses) of Janssen Vaccines' adenovirus‐vectored Ebola vaccine (Popova et al., 2016). Across Oxford, AZ and SII, the greatest previous scale of adenovirus vector manufacturing before the SARS‐CoV‐2 pandemic was <1 × 10^15^ VP (<20,000 doses) (Fedosyuk et al., 2019; O'Hara et al., 2012).

Adenovirus manufacturing typically involves amplification of master and working viral seed stocks which are then used to infect producer cells in a batch or perfusion upstream process (USP), followed by a multistep downstream process (DSP; most commonly depth filter clarification, tangential flow filtration [TFF], anion exchange [AEX] chromatography, and a second TFF step) (Vellinga et al., 2014). The complexity of such processes, relative to DNA and RNA vaccine platforms, has previously been considered disadvantageous for emerging pathogen response. ChAdOx1 nCoV‐19 faced potential additional manufacturing complexity in that the encoded “spike” antigen belongs to a protein class (viral fusion glycoproteins) which in our experience can sometimes inhibit cellular production of adenovirus.

We had previously designed a robust but small‐scale platform for production of multiple adenovirus vectors, including in response to emerging pathogen outbreaks (Fedosyuk et al., 2019). We have now developed a process with higher productivity and greater simplicity than that we had previously reported, and transferred the technology to multiple Good Manufacturing Practice compliant (GMP) production sites.

Here, we present the approach to and results of that development and technology transfer programme. In brief, our starting point was an observation in January 2020 of high productivity from a modified USP at 30 ml scale. In early February 2020, in light of the COVID‐19 outbreak in Wuhan, we demonstrated compatibility of this USP with our previous DSP at 3 L scale and began a programme of scale‐up which culminated in a proof‐of‐concept 200 L batch in April 2020. In parallel, we initiated technology transfer to multiple contract manufacturing organizations (CMOs). In the second quarter of 2020 we introduced process modifications, removing bottlenecks to enable commercial manufacturing at 1000–4000 L scale. The first 1000 L drug substance batch was completed in September 2020, and the billionth dose was released in July 2021.

## 2 METHODS

### 2.1 Viruses

The ChAdOx1 nCoV‐19, ChAdOx1 Lassa‐GP, ChAdOx1 luciferase, and ChAdOx2 GFP vectors used here have previously been described (Dicks et al., 2015; Morris et al., 2016; Purushotham et al., 2019; van Doremalen et al., 2020).

**Figure 1:**
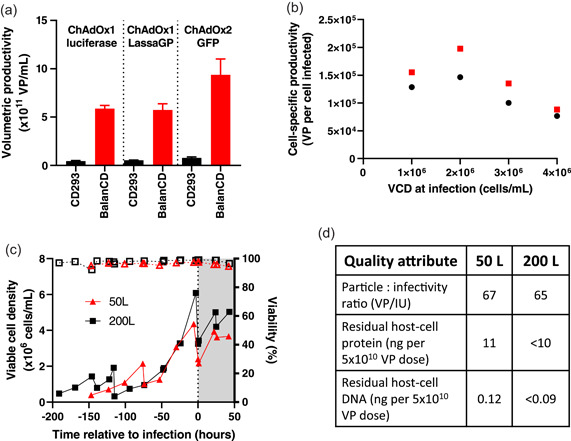
Development and scale‐up of fed‐batch process. (a) Small‐scale USP productivity of ChAdOx1 luciferase, ChAdOx1 LassaGP, and ChAdOx2 GFP with BalanCD medium/feed (infected at 4 × 10^6^ cells/ml, MOI = 10) as compared with our previously established conditions in CD293 medium (infected at 1 × 10^6^ cells/ml, MOI = 3) (Fedosyuk et al., 2019). ChAdOx1‐luciferase infections were performed in a 3 L bioreactor. The other two viruses were produced in 30 ml volume in shake flasks. Results shown are the median and range of qPCR results from technical duplicate samples from a single reactor for ChAdOx1‐luciferase, and from triplicate flasks for the other viruses. (b) Cell‐specific productivity of ChAdOx1 nCoV‐19 in shake flasks at 30 ml working volume at MOI = 3 (red) and MOI = 10 (black). For each condition, the peak volumetric productivity from the timecourse data shown in Figures S1b,c was converted to a cell‐specific productivity by division by the cell density at infection. (c) and (d) Examples of 50 and 200 L batches with high MOI fed‐batch upstream process. (c) Cell growth (solid lines) and viability (dashed lines). (d) Drug substance (DS) quality following purification. MOI, multiplicity of infection; qPCR, quantitative polymerase chain reaction; USP, upstream process

Virus used as seed to infect shake flask cultures (Figure 1a,b) and as standards in quality control assays (as stated in figure legends) was produced by caesium chloride density‐gradient ultracentrifugation by the Jenner Institute Viral Vector Core Facility.

**Figure 2:**
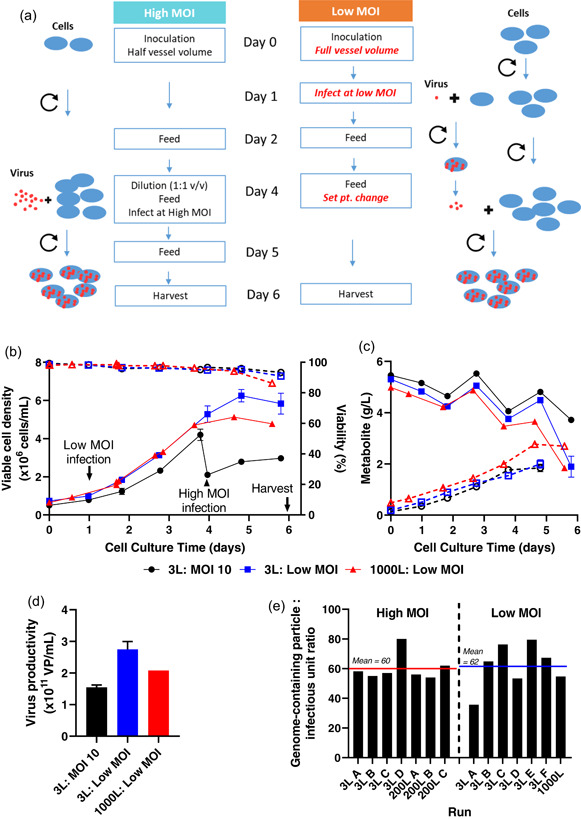
Development of a low MOI upstream process. (a) compares, in schematic form, the original high MOI USP with the low MOI process developed for scale up. Red text indicates key changes in the low MOI process. (b)–(d) Results with the low MOI upstream process at 3 L (blue) and 1000 L (red) scale, as compared with the MOI = 10 process at 3 L (black). (b) Cell counts (solid lines, filled symbols) and viability (dashed lines, open symbols). (c) Glucose (solid lines, filled symbols) and lactate (dashed lines, open symbols). (d) Productivity. Where present, error bars indicate median and range of duplicate cultures at 3 L scale. Other data was obtained in singlicate. (e) Similar genome‐containing virus particle: infectious unit ratios from successive runs of the high and low MOI processes at a variety of scales. qPCR and infectivity assays were performed on crude viral harvest samples collected 6 days after infection of the culture. MOI, multiplicity of infection; qPCR, quantitative polymerase chain reaction; USP, upstream process

Virus used as seed to infect bioreactor cultures before the development of the low MOI process (i.e., for experiments shown in Figures 1 and S1) was prepared using our previously described batch process in 3 L shake flasks or bioreactors, up to the point of the first tangential flow filtration (TFF) step (Fedosyuk et al., 2019). After this the concentrated and diafiltered lysate was aliquoted and frozen at −80°C. Virus used as seed in development of the low MOI process and at 1000 L scale (i.e., for experiments shown in Figures 2 and S3) was produced similarly, with the exception that that upstream process was performed at 200 L scale using the high MOI fed batch process described in this manuscript.

### 2.2 Cells and upstream process

A master cell bank derived from HEK293 cells expressing the *Escherichia coli* tetracycline repressor protein (Yao et al., 1998) was prepared and adapted to low‐serum suspension culture in CD293 medium (ThermoFisher) using methods as previously described (Fedosyuk et al., 2019). Cells were then adapted to increasing proportions of BalanCD293 medium (Fujifilm‐Irvine Scientific), supplemented with 4 mM GlutaMAX (ThermoFisher), over one week. Adaptation to other media (HyClone CDM4‐HEK293 [Cytiva] and Freestyle 293 [ThermoFisher]) was performed similarly. All feeds were with 0.05 volumes of BalanCD293 feed (Fujifilm‐Irvine Scientific), unless otherwise stated.

For upstream process experiments, seed culture at 2× the specified final density was diluted by addition of 1 volume of fresh medium to reach the cell density specified for each experiment at the point of infection. A multiplicity of infection of 10 was used unless otherwise stated.

Pre‐infection, cultures were fed on the day cell density exceeded 1 × 10^6^ cells/ml. Cultures for which the intended cell density at the point of infection was ≥3 × 10^6^ cells/ml received a second pre‐infection feed when cell density exceeded 4 × 10^6^ cells/ml. Postinfection, all cultures were fed at 0.5 and 22 h after infection.

Shake flask experiments were performed in Erlenmeyer flasks (Corning), with a working volume of 25–35 ml in a 125 ml flask unless otherwise stated. BioBlu 3c and 14c (Eppendorf) single‐use bioreactor vessels were used in accordance with the manufacturers' instructions. A GX bioreactor controller unit and C‐BIO software (both from Global Process Control) were used to control both vessel types. Dissolved oxygen (DO) was regulated at a setpoint of 55% air saturation by addition of medical air via macrosparger. pH was regulated in the range 7.2–7.3 as previously described (Fedosyuk et al., 2019).

50 and 200 L upstream processes were performed using Pall Allegro™ stirred tank reactors (STRs). For work using the high MOI fed batch process (shown in Figure 1c,d), bioreactors were seeded at 0.4–0.6 × 10^6^ cells/ml in c. 35% of the maximum working volume. Antifoam C emulsion (SigmaAldrich) was used in 50 and 200 L STRs. 0.05 culture volumes of BalanCD feed was added when the density reached 1.0 × 10^6^ culture cells/ml. At a cell density of 4.0 × 10^6^/ml (range 3.0–6.0 × 10^6^), cells were diluted with 1 volume of medium and infected, using an MOI of 10 unless otherwise stated. 0.05 volumes of BalanCD feed were added 30 min after infection, and again after 22 h.

For the low MOI upstream process (Figure 2), bioreactors were seeded at 0.7 × 10^6^ cells/ml in c. 70% of the maximum working volume. 16 to 28 h after inoculation, the cells were infected at an MOI in the range 0.025 to 0.4. Two BalanCD feeds (5% v/v) were added to cell culture at 48 ± 4 and 96 ± 4 h post bioreactor inoculation. Bioreactor temperature was reduced to a lower set point within 4 h of the second feed. About 140 h postinoculation, the cell culture was harvested and processed for analysis.

### 2.3 Lysis, nucleic acid digestion, and clarification

Lysis was performed as previously described (Fedosyuk et al., 2019), in the culture vessel, with the exception that the concentration of Benzonase (MerckMillipore) was reduced to 15 units/ml. Lysis was initiated at 42–48 h after infection, with the exception of the productivity kinetic experiments shown in Figure S1b,c. Two hours after addition of lysis buffer, clarification was initiated, using Millistak+® HC Pro C0SP depth filters as in our previous work (Fedosyuk et al., 2019). During 200 L runs, an Allegro™ Advanced MVP skid (Pall Biotech) was used for filtration steps.

### 2.4 Tangential flow and bioburden reduction filtration

Tangential flow filtration was performed essentially as we have previously described (Fedosyuk et al., 2019), scaled appropriately and with the following modifications. Where TFF was performed before AEX, that is, for the 200 L run producing product as reported in Figure 1c,d, only twofold concentration was performed, before 6 diavolumes of diafiltration. For TFF after AEX, Omega™ T‐series 300 kDa cut‐off flat sheet filters (Pall Biotech) were used. For TFF during 200 L runs, an Allegro™ CS 4500 single‐use TFF skid was used (Sartorius). A Supor® EKV 0.2 μm filter was used for bioburden reduction filtration after the final TFF.

### 2.5 AEX chromatography

Where preceded by TFF (run reported in Figure 1c,d), AEX was performed as previously reported (Fedosyuk et al., 2019), with scaling of the chromatography capsule and buffer volumes based upon anticipated binding capacity of 7×10^13^ VP per mL of membrane volume.

For “direct‐load” AEX (loading clarified lysate), the small‐scale studies shown in Figure S2a–c were performed using an Akta Pure instrument (Cytiva) and 3 ml bed volume/8 mm bed height Sartobind Q Nano capsules (Sartorius). Equilibration buffer comprised 20 mM Tris‐HCl, pH 8.0, 1 mM MgCl_2_, 0.1% v/v polysorbate 20, 5% w/v sucrose. Elution buffer comprised 20 mM Tris‐HCl, pH 8.0, 1 mM MgCl_2_, 0.1% v/v polysorbate 20, 5% w/v sucrose, 600 mM NaCl, except where salt concentration was varied, as stated. Wash buffers of the desired conductivities for each experiment were prepared by mixing equilibration and elution buffers. Adjustment of the conductivity of the sample, to target values as stated in the descriptions of individual experiments, was performed using 5 M NaCl (Sigma). Column equilibration was in accordance with the manufacturer's instructions. After loading, capsule was washed with 10 membrane volumes (MV) of equilibration buffer before the elution step (both at 5 MV/min).

For the “direct‐load” AEX purifications from a 10 L bioreactor (Figure S2e,f) a peristaltic pump‐driven rig was constructed, as shown in Figure S1e, incorporating a C0SP depth filter (as above), Millipak‐20 0.2 μm filter, and 150 ml/8 mm bed height Sartobind Q capsule (Sartorius), plus single‐use UV absorbance, conductivity and pressure sensors (Pendotech). Buffers, column equilibration, sample loading, washing and elution were as described above, with the exceptions that wash buffer was prepared by addition of 5 M NaCl to the equilibration buffer, and a flow rate of 0.7 membrane volumes/minute was used for sample loading, washing and elution.

**Figure 3:**
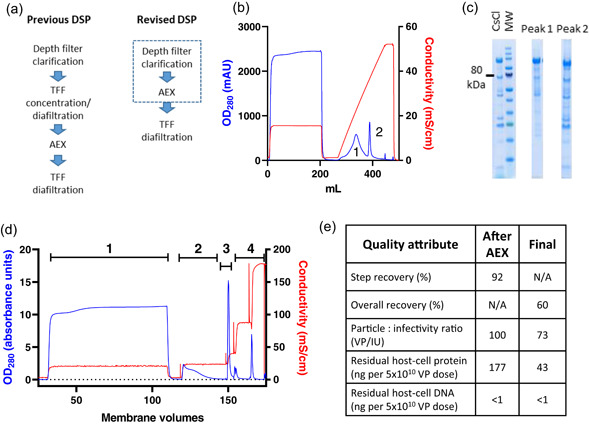
Simplified downstream process, with direct loading of clarified lysate on AEX. (a) Schematics of previous and revised DSPs. The dashed box indicates potential execution of depth filter clarification and AEX as a single unit operation. (b) Loading of clarified lysate on 3 ml Sartobind Q anion exchange membrane, followed by elution with a gradient of increasing salt concentration. The peaks labeled 1 and 2 (at 24 and 37 mS/cm) were analyzed by Coomassie‐stained SDS‐PAGE (c), with comparison with virus purified by caesium chloride gradient ultracentrifugation (CsCl) and molecular weight marker (MW, with 80 kDa indicated). Peak 1 contains impurities (notably free hexon protein) while Peak 2 contains predominantly virus. (d) AEX chromatogram obtained using clarified lysate from low MOI upstream process, run at 1000 L scale. Absorbance at 280 nm is shown in blue, conductivity in red. Results are shown from one of two cycles run on a 5000 ml Sartobind Q capsule, each loaded to approximately 1.5 × 10^13^ VP per ml of membrane. Numerals indicate stages: 1 = loading, 2 = wash, 3 = elution, 4 = 1 M NaCl strip and 1 M sodium hydroxide sanitization. (e) Product recovery and quality from the 1000 L scale process shown in (b), after AEX and after final formulation by TFF and 0.2 μm filtration. AEX, anion exchange; DSP, downstream process; MOI, multiplicity of infection; SDS‐PAGE, sodium dodecyl sulfate–polyacrylamide gel electrophoresis; TFF, tangential flow filtration

For the “direct‐load” AEX purification from a 1000 L bioreactor (Figure 3b) a PK50 liquid chromatography skid (Sartorius) and 5000 ml/8 mm bed height Sartobind Q capsule (Sartorius) was used. Column equilibration was in accordance with the manufacturer's instructions. Equilibration buffer comprised 50 mM Tris‐HCL pH 8.0, 1 mM MgCl_2_, 5% w/v sucrose. After loading, the capsule was washed with 10 MV of equilibration buffer followed by at least 20 MV of wash buffer (50 mM Tris‐HCl, pH 8.0, 222 mM NaCl, 1 mM MgCl2, 5% w/v sucrose). Product was eluted with 5 MV of elution buffer (50 mM Tris‐HCL pH 8.0, 444 mM NaCl, 1 mM MgCl_2_, 5% w/v sucrose). Loading, equilibration, washing, and elution were all performed at 2.5MV/min.

Small‐scale studies shown in Figure S3 were performed as described for Figure S2, with the exception that the buffers used were as described for the 1000 L run were used.

### 2.6 Product quantification and assessment of product quality

Product quantification was as previously reported, using quantitative polymerase chain reaction (qPCR) and UV spectrophotometry assays for viral particles in impure and pure samples, respectively, and an immunostaining‐based infectivity assay (Fedosyuk et al., 2019). In addition to replication described in the figure legends, technical triplicate reactions/readings were performed for all qPCR and UV measurements.

qPCR data shown in Figure 2 was produced using an assay with some modifications from that which we previously described. Primers and probe were specific to the spike protein transgene rather than the adenovirus backbone (CTGGATCCTCTGAGCGACAC, TGGTAGATGCCCTTTTCCAC and 5′ 6‐FAM/AAGTGCACC/ZEN/CTGAAGTCCTTCACC 3′ ABkFQ [Integrated DNA Technologies Inc.]). Samples were pretreated with 50 U/ml DNase I (ThermoFisher) at 37°C for 15 min to remove unencapsidated DNA, followed by addition of EDTA to 50 mM final concentration to halt DNase activity, lysis of the resulting sample (by 1:1 mixing with buffer comprising 0.2% SDS, 50 mM EDTA, 0.2% Triton‐X100, 400 μg/ml proteinase K, and incubation at 56°C for 15 min), and finally 1:9 dilution in 10 mM Tris/1 mM EDTA pH 8.0.

Residual host–cell protein (HCP) was quantified using the HEK293 HCP ELISA kit (Cygnus Technologies), according to the manufacturer's instructions. Residual host cell DNA was quantified using a previously reported quantitative PCR method targeting a 94 base pair amplicon within the Alu repeats (Zhang et al., 2014). The lower limit of quantification was 100 pg/ml for intact HEK293 DNA.

### 2.7 Process economic modeling

Costs were evaluated using Biosolve Software (BiopharmaServices). A detailed execution protocol for drug substance production (from seed vial to final sterile filtration) was combined with mass balance data as described in this manuscript to build and parametrize a model. Assumptions made were designed to be conservative: productivity of 2 × 10^14^ VP/L; a requirement for 7.5 × 10^10^ VP of drug substance to provide one extractable therapeutic dose of 5 × 10^10^ VP; and a facility with a single 2000L bioreactor and utilization of 70% providing 32 batches per year, operating with a three‐shift upstream and two‐shift downstream production shifts.

## 3 RESULTS

### 3.1 Initial development of a fed batch upstream process for adenovirus production

We initially investigated medium/feed combinations for a fed‐batch USP. In preliminary work, we compared cell growth and viral productivity across a range of cell densities in a variety of commercially available media (Figure S1a). Of these, BalanCD HEK293 medium and feed (Fujifilm) was found to support growth of vaccine‐antigen‐repressing producer cells (see Supporting Information) to 1.2 × 10^7^ cells/ml with high viability (Figure S1a). Using this combination in small‐scale production of adenovirus vectors of two serotypes and carrying three transgenes, we attained productivity exceeding 5 × 10^11^ virus particles (VP) per ml, around fivefold greater than typically obtained in our previous USP (Figure 1a). To our knowledge such productivity has not previously been reported from a non‐perfusion USP.

Upon availability of ChAdOx1 nCoV‐19 starting material, we assessed productivity with varying multiplicity of infection (MOI), cell density and time of harvest (Figures 1b and S1b,c). Although productivity was somewhat lower than seen with the previously tested vectors, it remained favorably comparable to previously reported processes. Productivity of non‐perfusion adenovirus processes is limited by the so‐called “cell density effect”: falling cell‐specific productivity occurring within the range of cell densities at which exponential growth can be maintained. A fall in cell‐specific productivity to <1 × 10^5^ VP/cell is commonly observed at cell densities exceeding 1 × 10^6^ cells/ml (Kamen & Henry, 2004). Our chosen medium/feed regime maintained good cell‐specific productivity at up to 2 × 10^6^ cells/ml but the effect remained clear at higher densities (Figure 1b).

We have previously demonstrated and discussed advantages of the use of a cell line/promoter combination which represses antigen expression during production (Cottingham et al., 2012; Fedosyuk et al., 2019). In the case of ChAdOx1 nCoV‐19 (bearing the spike transgene under a repressible promoter) we did not see a consistent effect of antigen repression upon viral productivity. Nonetheless we proceeded with use of an antigen‐repressing cell/promoter combination on the basis of previous observations that this can reduce risk of emergence, during serial passage of the virus, of mutations abrogating antigen expression.

An MOI of 5–10, cell density of 2–3 × 10^6^/ml and time of harvest 42–48 h postinfection were selected for scale up. USP productivities in 10, 50, and 200 L stirred tank reactors (STR) remained in the range 2–4 × 10^11^ VP/ml, with acceptable cell growth and metabolite profiles (Figures 1c and S1d).

Early STR batches made use of a DSP very similar to that we had previously described, which again achieved recovery of 50%–60% and quality characteristics compliant with a regulator‐accepted specification for product for clinical use (Figure 1d) (Fedosyuk et al., 2019).

### 3.2 A low MOI upstream process enables economical use of virus seed

Due to the scale of the pandemic, the need to further scale the process to large numbers of 1000‐2000L batches was apparent. This posed challenges including the provision of adequate virus seed stock, volumes of buffer to be handled in the downstream process, and supply of materials.

The “amplification factor” (output virus as a multiple of input seed) of a high MOI species E adenovirus production process is relatively low. Considering the high particle:infectivity ratio of these vectors (typically c. 70), after estimating likely losses in processing and aliquoting and adding a margin for safety, we felt we could only rely on production of c. 300 IU of useable seed per cell infected during seed production: this would be sufficient to infect only 30 cells at an MOI of 10. This 30‐fold amplification factor implied the need for large numbers of batches of working virus seed (creating a testing burden and consistency concerns), and a possible requirement for multi‐step seed expansion (from master virus seed through two working seed production passages). Reduction of the MOI to c. 3 was considered, but we felt this would be unlikely to fully address these concerns.

To avoid the complexity arising from a high seed requirement, we investigated use of MOI < 1. By infecting cells at low density within 24 h of bioreactor inoculation with cells, and then continuing culture for a further 120 h, we allowed the initially‐uninfected cells to proliferate before becoming secondarily infected by the released virus particles from first round of infection (Figure 2a). Informed by a preliminary experiment comparing a range of MOIs <1 in small‐scale parallel bioreactors (Ambr250, Sartorius), we selected an optimal MOI, balancing process duration, consistency and productivity. Use of this MOI at 3 L scale maintained or improved volumetric productivity as compared to the original MOI = 10 process; these results were closely replicated by a subsequent low MOI run at 1000 L scale (Figure 2b–d). Product quality (as assessed by genome copy: infectious unit ratio) was similar from the high MOI and low MOI processes across a range of scales (Figure 2e).

The reduced MOI, and resulting increased virus amplification factor, has enabled seed from a single 200 L bioreactor batch to supply global manufacturing needs.

### 3.3 Chromatography fed directly with clarified lysate facilitates large‐scale purification

The initial TFF step was identified as the key bottleneck for DSP scale‐up. Time did not permit extensive process characterization before scale‐up, and we were concerned that excessive concentration of the lysate during TFF would increase risk of formation of aggregates of product with host‐cell protein and DNA. Using only modest concentration to favor process robustness meant that multiple process volumes of diafiltration buffer and waste needed to be handled, which we anticipated would be challenging in some facilities. The original process also required large TFF membrane areas (c. 1 m^2^ per 40 L of lysate) and although we anticipated this could be reduced with optimization, we were concerned that TFF membrane supply could become problematic due to high demand during the COVID‐19 pandemic.

We therefore sought to develop a simplified DSP by loading the clarified lysate directly on an AEX membrane, followed by a single diafiltration polish/formulation step. As well as eliminating a process step, this process change provides the option of execution of clarification and AEX as a single unit operation (Figure 3a), although we have not yet pursued this possibility at large scale. Following small‐scale optimization (Figures 3b,c and S2a–c), we executed the complete revised DSP at 10 L and then 50 L scale (Figure S2d–f). With feed from the low MOI USP, increased concentrations of host‐cell‐derived impurities reduced the AEX binding capacity to ~1.5 × 10^13^ VP per mL of membrane, but we demonstrated re‐use of the membranes up to 20 times without deterioration in product recovery or purity, and robustness of the process at a wide range of flow rates (Figure S3).

We proceeded to implement this process at 1000–4000 L scale. AEX step recovery at >1000 L scale is typically >80%, and after a final TFF step and bioburden reduction filtration, overall downstream process recovery is typically 60%–65% (Figure 3d,e).

Quality of product from this process was within acceptable limits (Figure 3e). Comparing to the quality of product from the original process (incorporating pre‐AEX TFF, as exemplified by the data in Figure 1h), the viral particle: infectivity ratios from the two processes were essentially indistinguishable (these values are calculated from the results of two separate assays, each with a margin of error). P:I ratio specifications have tightened as our programme has progressed, but these values <100 would comply with all specifications which have been applied. Residual host cell DNA in product from both processes was beneath the lower limit of detection of our assay, and <1/10 of the widely accepted upper limit of 10 ng/dose (Yang, 2013). Residual host cell protein levels are somewhat higher in product from the direct‐AEX process, but the value of 43 ng/dose obtained here remained well within limits which have been accepted for other vaccines made on human cell lines (Institute for Vaccine Safety, n.d.).

### 3.4 Technology transfer for rapid, large‐volume, economical distributed manufacturing

From early 2020, we were conscious that manufacturing, rather than clinical trials (or, in most places, vaccine distribution), was likely to prove limiting for the speed of global COVID‐19 vaccination rollout. We were also concerned that so‐called vaccine nationalism was likely to impede equitable access to effective vaccines, as had been seen during the H1N1 swine flu pandemic (Fidler, 2010).

We therefore set out to design a global manufacturing strategy to achieve three goals: low cost; speed to large volume supply, including prompt wide geographical availability; and, critically, consistent high quality.

**Figure 4:**
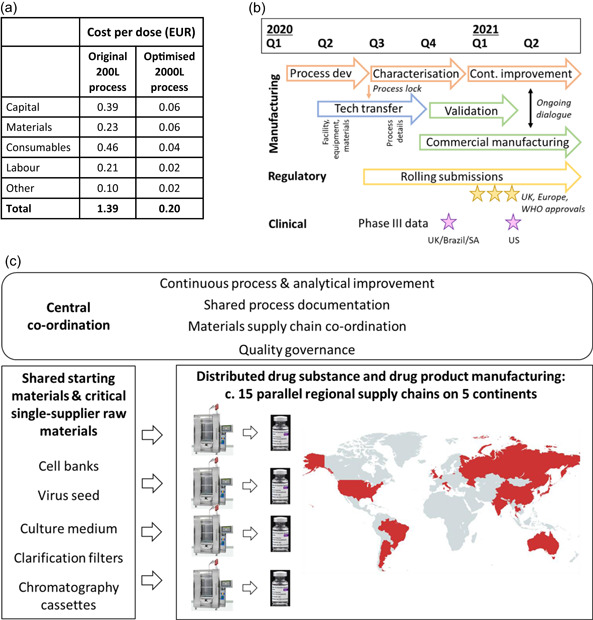
Rapid implementation of a low cost distributed manufacturing strategy. (a) tabulates modeled costs of bulk DS production using the initial process at 200 L scale (with high MOI and including the additional TFF step), and using the optimized process at 2000L scale (with low MOI and direct AEX). This excludes fill/finish and some analytical costs. For further details, please see Supporting Information. (b) A timeline of key manufacturing‐related activities, highlighting activities performed in parallel and relationship to the timing of key regulatory and clinical events. (c) illustrates global distributed manufacturing strategy, with interplay between centrally co‐ordinated activities, common origins of certain key materials, and multiple parallel regional drug substance and drug product supply chains. ChAdOx1 nCoV‐19 drug substance is currently being manufactured in the countries shown in red. Vial photograph: Arne Müseler/arne‐mueseler.com/CC‐BY‐SA‐3.0/https://creativecommons.org/licenses/by-sa/3.0/de/deed.de. Map created using mapchart.com, under CC‐BY‐SA‐4.0 licence. AEX, anion exchange; DS, drug substance; MOI, multiplicity of infection; TFF, tangential flow filtration

Low cost was achieved due to the high productivity of the process and the use of off‐the‐shelf equipment and materials. Modeling of DS manufacturing using a commercial cost‐modeling package suggests a cost of goods of <EUR 1 per dose (Figure 4a). This forecast does not account for geographical variation in input costs, nor non‐DS costs such as vial filling, research or regulatory expenses. The model suggests that the cost of goods of DS is relatively low and, hence, these other costs contribute the majority of the cost of the finished product.

Despite the productivity of the process, it was apparent multiple manufacturing facilities were required to achieve speed to large volume supply: global supply required >1 m litres of upstream process output and hence a large number of batches. Moreover, location of DS manufacturing appeared likely to be a key determinant of vaccine availability, especially for low and middle income countries. From the outset (Q1 2020), our strategy therefore included simultaneous technology transfer to multiple sites, including in India and China. Through Q2‐3 2020, this strategy was further developed, resulting in distribution of drug substance manufacturing across a network of existing contract manufacturing and licensee sites capable of single‐use bioreactor (SUB) viral manufacturing at 1000–4000 L scale (Figure 4b,c).

To achieve the earliest possible release of commercial product through this distributed manufacturing strategy, we “parallel‐tracked” a number of process development, tech transfer, validation and regulatory activities which would more typically run sequentially (Figure 4b). For several manufacturing sites, aspects of technology transfer (e.g., equipment and materials procurement/validation testing) began before process development was completed and the process was “locked” for regulatory submissions. Initial regulatory submissions for emergency use approval occurred in parallel with process performance qualification (PPQ) runs at the initial manufacturing sites. Simultaneous initiation of manufacturing at multiple sites during a pandemic which impeded travel (and hence site visits) posed obvious challenges in maintaining product quality and consistency. The technology transfer process therefore used shared documents (e.g., process description) to the greatest extent possible, and incorporated a robust quality assurance strand and measures to ensure cross‐site analytical comparability. We conducted intensive process characterization after the start of commercial production, involving both sampling from large‐scale manufacturing at multiple sites and ongoing work with small‐scale process models in a central laboratory. This led to the identification of opportunities for optimization to improve process control and the quality and quantity of output, within the parameters of the approved and validated process: these included improvements in control of the cell seed train, cell lysis protocol, and DSP intermediate hold steps. Figure 4b–d depict the ongoing interplay of central co‐ordination and optimization with production at the multiple sites.

## 4 DISCUSSION

Vaccine supply is proving to be the key determinant of timing of COVID‐19 vaccination, especially in low and middle‐income countries.

Some aspects of adenovirus‐vectored vaccine manufacturing are inherently well‐suited to pandemic response. Antigen‐repressing promoter/cell combinations, and the absence of the encoded antigen from the adenovirus virion, enable similar upstream, downstream and drug product manufacturing regardless of the encoded antigen, and we had previously described a small‐scale process designed for rapid response to emerging pathogens (Fedosyuk et al., 2019). Until 2020, however, adenovirus vectors had only been manufactured at scales orders of magnitude smaller than necessary for pandemic response. Adenovirus manufacturing (which requires a mammalian cell‐based, rather than microbial or synthetic approach) has been perceived by some to be relatively complex and hence potentially slower than nucleic‐acid‐based alternatives. We therefore sought, from early 2020, to develop production technology which could address challenges of speed, scale, and equitable access.

Our initial priority was to achieve sufficient volumetric productivity of the upstream process: a chosen medium/feed regime enabled the maintenance of cell‐specific productivity at relatively high cell density. We then sought to resolve two problems which we anticipated would hinder very large‐scale production. First the fed batch USP was coupled with use of a low MOI two‐viral‐lifecycle process: this reduced working virus seed requirements >30‐fold. Second, direct loading of clarified lysate on AEX enabled removal of a TFF step from the DSP. Validation of flexible chromatography conditions (including membrane re‐use and variable flow rates) facilitates the implementation of the process using standard equipment available at most sites, reduced material requirements, and achieved chromatography cycling times as short as 2 h at 2000 L scale. Efficient recovery and high quality of adenovirus from such a “direct load” AEX has not to our knowledge previously been reported.

Taken together, these process improvements have allowed production campaigns at 1000–4000 L scale at multiple sites. Typical yields are comfortably more than 1000 usable doses of drug product (each 5 × 10^10^ VP) per litre of upstream culture. We believe this productivity is around double that of previously disclosed batch or fed batch processes for adenovirus manufacture (Nadeau & Kamen, 2003; Shen et al., 2016), and the resulting cost of goods of the drug substance is <EUR 1/dose.

This process is not unique either in the scale of individual batches, or in its volumetric productivity: we are aware both of previous manufacturing of individual 1000 L batches of adenovirus vectors, and of more complex perfusion‐based adenovirus production processes which can achieve volumetric productivity in the region of 1 × 10^15^ VP per litre (Popova et al., 2016). Instead, we believe the advance this process offers is the combination of moderately high productivity with the simplicity to permit global scale‐out of multi‐batch campaigns, and hence to maximize total output.

Our process development prioritized simplicity to enable execution in as many existing facilities as possible. We used single‐use product‐contact materials throughout and unit operations which are common across the bioprocess industry (although dissimilar to a traditional viral vaccine process, adenovirus production has similarities to a modern mammalian‐cell‐based therapeutic protein manufacturing process). Combined with the biosafety of the vector (BSL1‐2 dependent upon jurisdiction), the design of the process enabled a distributed manufacturing strategy, with several independent national or regional supply chains. Such strategies have previously been proposed as means of ensuring wide geographical availability of vaccines, but never to our knowledge implemented with this speed and scale. In the context of current calls for waivers of intellectual property relating to COVID‐19 vaccines, it is important to note the degree of central co‐ordination which has been necessary to ensure efficient manufacture and consistent quality across the multiple production sites (Figure 4b,c).

The process described here has thus provided a substantial proportion of global COVID‐19 vaccine supply to date (>1 billion doses released for supply as of August 2021). Uniquely among current COVID‐19 vaccines from high‐income country developers, the programme has aimed to achieve “equity by design”: as a result of the process development and technology transfer strategy, the majority of output has been manufactured and/or used in low/middle income countries.

## CONFLICT OF INTERESTS

C. C. D. J., Y. L., S. F., G. G., A. B., R. R. S., Te. L., S. C. G., A. J. R., and A. D. D. are named inventors or contributors to intellectual property assigned to Oxford University Innovation relating to the ChAdOx1 nCoV‐19 vaccine and/or manufacturing process, and may receive a proportion of proceeds from out‐licensing of the intellectual property. J. J., Th. L., N. B., D. P., R. V., and R. T. are employees of AstraZeneca.

## AUTHOR CONTRIBUTIONS

*Conceptualization*: Alexander D. Douglas and Richard Turner. *Experimental design, planning, execution, and data acquisition*: Carina C. D. Joe, Jinlin Jiang, Teresa Lambe, Yuanyuan Li, Sofiya Fedosyuk, Gaurav Gupta, Adam Berg, Rameswara R. Segireddy, David Mainwaring, Amar Joshi, Paul Cashen, Byron Rees, Jonathan Humphreys, Sarah Davies, Nick Smith, Scott Bruce, Dennis Verbart, Daan Bormans, Carol Knevelman, Mark Woodyer, Lee Davies, Lisa Cooper, Maria Kapanidou, Nicole Bleckwenn, Daniel Pappas, Daniel C. Smith, Catherine M. Green, Raghavan Venkat, Adam J. Ritchie, Richard Turner, and Alexander D. Douglas. *Construction of process model*: Nitin Chopra and Piergiuseppe Nestola. *Provision of critical materials*: Teresa Lambe and Sarah C. Gilbert. *Funding acquisition*: Sarah C. Gilbert, Richard Turner, and Alexander D. Douglas. *Writing and review of manuscript*: all authors.

## Supporting information

Supporting information.

## Data Availability

The data sets generated during the current study are available from the corresponding author on reasonable request.
