# Supplementary material for: Manufacturing a chimpanzee adenovirus‐vectored SARS‐CoV‐2 vaccine to meet global needs
Source: Biotechnol Bioeng. 2021 Nov 15;119(1):48–58. doi: 10.1002/bit.27945 (PMC8653296; doi:10.1002/bit.27945)
Supplement: Supplementary file 1 — Supporting information. [file BIT-119-48-s001.docx]

Supplementary material: Manufacturing a chimpanzee adenovirus-vectored SARS-CoV-2 vaccine to meet global needs

## Supplementary Figures

### Supplementary Figure 1: Development of high MOI fed-batch upstream process


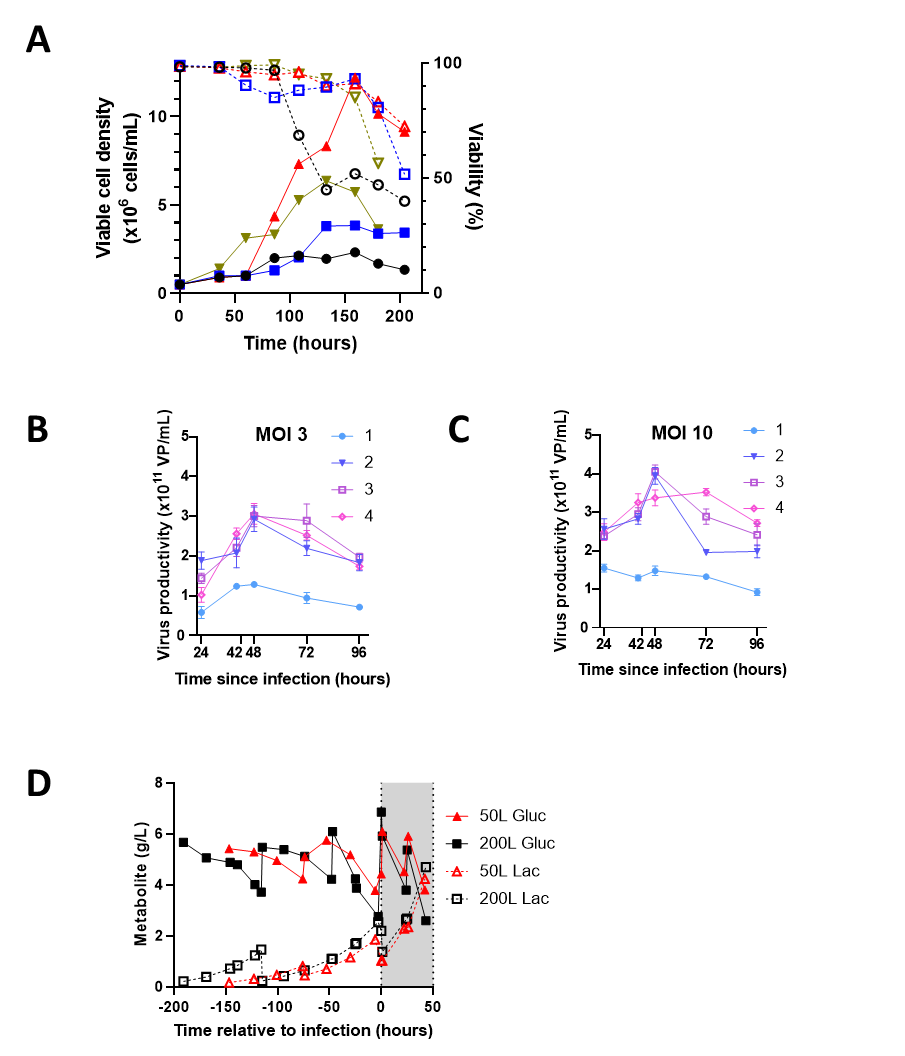


*Panel A shows cell counts (solid lines, filled symbols) and viability (dashed lines, open symbols) attained during growth of producer cells in BalanCD HEK293 medium without feeding (blue) or with addition of BalanCD HEK293 feed (red; 5% v/v at 36 and 108 hours), as compared to the CD293 medium (black, Thermo) used in our previous process. The plot also shows the next-best performing medium-feed combination from our evaluation (HyClone CDM4-HEK293 [Cytiva] plus BalanCD HEK293 feed, olive). Growth was also evaluated in Freestyle293 medium (ThermoFisher) and with a proprietary feed supplement, both of which performed inferiorly.*

*Panels B-C show small-scale USP productivity of ChAdOx1 nCoV-19 in shake flasks at 30mL working volume at MOI=3 (C) and MOI=10 (D) respectively. Legend indicates cell density represented by each line, in million cells/mL at point of infection. Infectious unit (IU) titres broadly paralleled VP titers; results are representative of two replicate experiments. Points indicate median and error bars show range of qPCR results for 2-3 replicate flasks.*

### *Panel D: Glucose (solid lines) and lactate (dashed lines) concentrations during 50L and 200L batches shown in Figure 1C.*Supplementary Figure 2: Optimisation of anion exchange with direct loading of clarified lysate


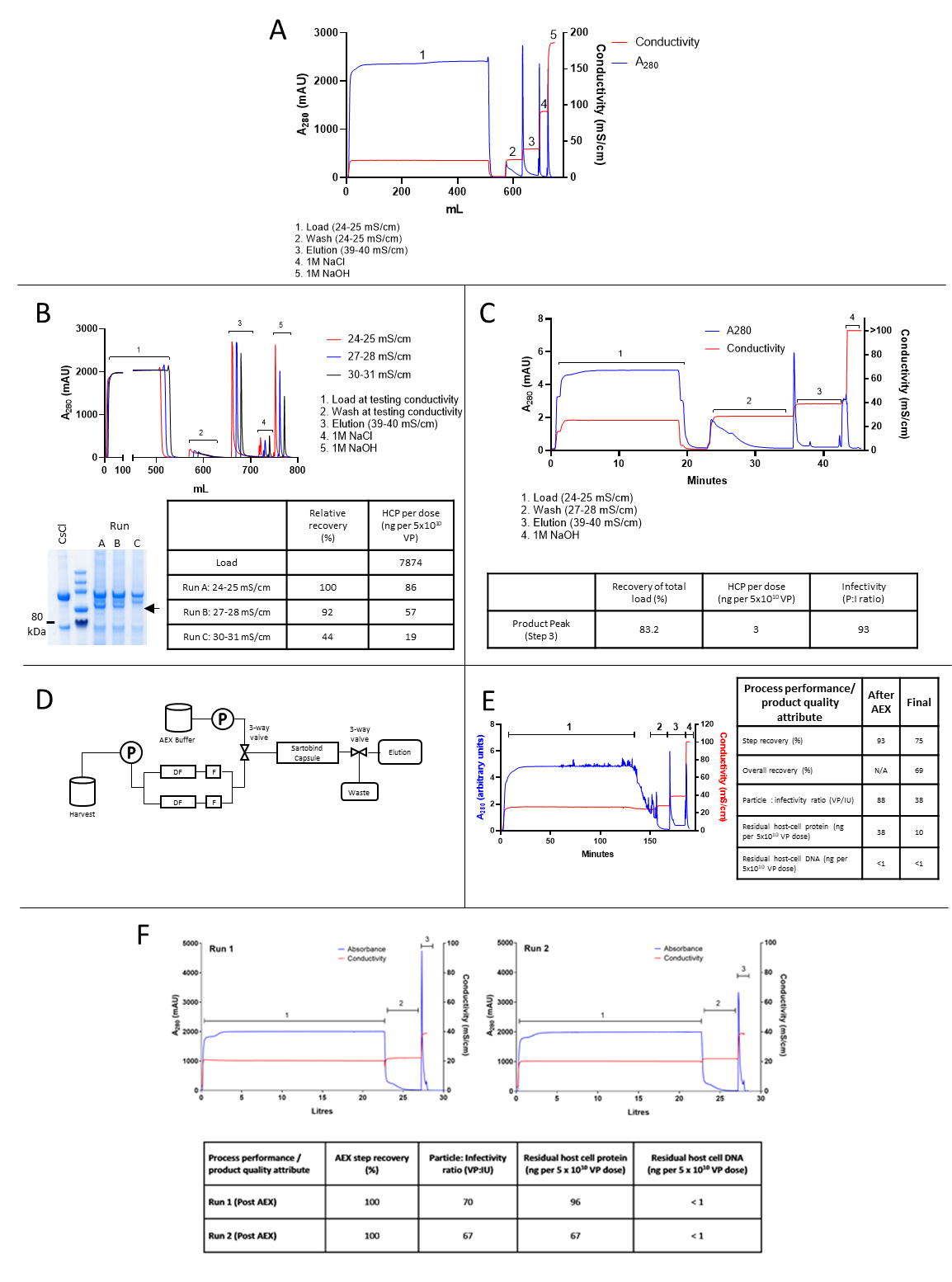


Supplementary Figure 2: Optimisation of anion exchange with direct loading of clarified lysate

Panel A shows initial estimation of binding capacity and product recovery by step elution. Clarified lysate containing 2x10^14^ VP of ChAdOx1 nCoV-19 was loaded onto a 3mL Sartobind nano Q capsule. Binding capacity and quantity of product bound was determined by collection of serial fractions of flowthrough during loading (step 1). After washing with equilibration buffer (step 2), product was eluted with a step to 39-40 mS/cm (step 3); steps 4 and 5 indicate regeneration with 1M NaCl and 1M NaOH respectively. Flowthrough and elution fractions were analysed by qPCR to calculate binding capacity and recovery: 10% breakthrough occurred at a load of 3.4x10^13^ VP per mL of bed volume; 90% of bound product was recovered.

Panel B shows optimisation of salt concentration / conductivity during loading and washing. Three runs were performed, in each of which filtered lysate containing 2x10^14^ VP of ChAdOx1 nCoV-19 was loaded onto a 3mL Sartobind nano Q capsule, after adjustment of conductivity to the indicated values by addition of salt. Each run used wash buffer with conductivity matching the load. The chromatogram overlays results from the three runs. Eluates were analysed by Coomassie-stained SDS-PAGE, qPCR and HCP ELISA. Recovery and HCP are tabulated: the ‘basal’ condition (24-25 mS/cm) achieved nearly 2-log_10_ reduction in HCP; increasing the conductivity of the load and wash achieved modest improvement in HCP clearance, with substantially reduced recovery with loading at 30-31 mS/cm. Recovery is shown relative to that achieved with the ‘basal’ condition.

Panel C shows initial results obtained using a 150mL Sartobind Q capsule with relatively low load challenge. Clarified lysate containing 7.4x10^14^ VP of ChAdOx1 nCoV-19 was loaded, using conditions as indicated, based upon the results of the experiment shown in Panel C. The eluate was analysed by qPCR, HCP ELISA and infectivity assay. Recovery as % of loaded product, HCP and P:I ratio are tabulated.

Panels D-E: in-line clarification and AEX at 10L scale. (D) illustrates *system used for in-line clarification and anion exchange (P: pump; DF: depth filter; F: 0.2µm filter).* (E) shows AEX chromatogram. Absorbance at 280nm is shown in blue, conductivity in red. A 150mL Sartobind Q capsule was loaded at 3.8x10^13^ VP per mL of membrane. Numerals indicate stages: 1 = loading, 2 = wash, 3= elution, 4= 1M sodium hydroxide sanitisation. Inset table shows product recovery and quality from AEX and after final formulation by TFF.

Panel F shows initial AEX performance data for two independent studies conducted using material generated from a 50 L production bioreactor. In each study, a total volume of 22.5 L of clarified harvest material was loaded onto a single 150 mL Sartobind Q capsule and subsequently eluted using a buffer of conductivity 39 – 40 mS/cm. AEX chromatograms for both studies (Run 1 and Run 2) are presented and relevant AEX process steps are indicated: (1) = loading, (2) = wash and (3) = elution. Insert table shows product recovery, and quality attributes from each run, immediately after the AEX step. The results demonstrate complete product recovery over the AEX step and similar process performance across the two studies.

### Supplementary Figure 3: Sartobind Q cycling and flow rate studies using feed stock from low-MOI process


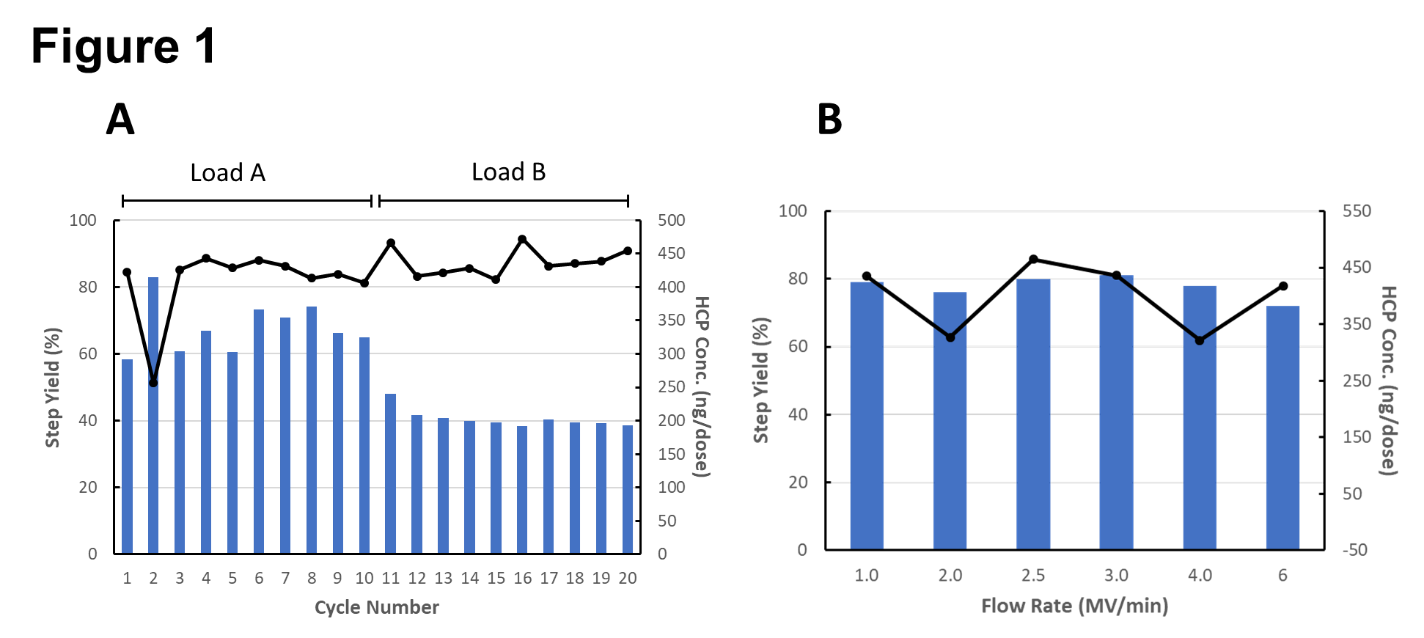


Panel A shows 20 cycles on 3mL Sartobind Q capsule. After each cycle, the membrane was stripped and sanitized. Before each cycle the membrane was activated with 1M NaCl and equilibrated. Two different load materials were used for this study (Load A and Load B).

Panel B shows process performance and product quality achieved on 3mL Sartobind Q capsule under baseline conditions with different flow rates. The flow rates shown in panel B were used for equilibration, loading, washing and elution steps.

For both A and B, Sartobind Q step yield is shown in black line graph. HCP concentration per dose in Sartobind Q product is shown in blue bar graphs (note that this precedes TFF).
